# Bedaquiline resistance: a predictable consequence of a game-changing drug?

**DOI:** 10.1128/asmcr.00228-25

**Published:** 2026-05-04

**Authors:** Ronald Lubelchek

**Affiliations:** 1 Cook County Department of Public Health 6144, Forest Park, Illinois, USA; 2 Division of Infectious Diseases, John H. Stroger, Jr. Hospital of Cook County 25430, Chicago, Illinois, USA; Rush University Medical Center, Chicago, Illinois, USA

## Abstract

Bedaquiline’s (BDQ) incorporation into the recommended treatment for drug-resistant tuberculosis (DR-TB) has ushered in a new era of highly effective, shorter course, all-oral regimens. Unfortunately, as the uptake of BDQ-based regimens has increased, so has the emergence of BDQ resistance. In a recent *ASM Case Reports* article, M. Richard-Greenblatt, R. Bagga, C. Duncan, M. J. Billick, et al. (ASM Case Rep 2:e00126-25, 2025, https://doi.org/10.1128/asmcr.00126-25) reported on a patient with treatment-emergent extensively drug-resistant (XDR) TB, whose isolate harbored a novel loss-of-function *pepQ* mutation, with resulting BDQ resistance. This commentary briefly highlights the benefits of BDQ-based DR-TB therapy, while also reviewing data related to the growing challenge posed by BDQ resistance.

## COMMENTARY

The use of bedaquiline (BDQ)-based regimens has revolutionized the management of drug-resistant tuberculosis (DR-TB). As the uptake of BDQ, a diarylquinoline antimicrobial, has risen, concern has grown regarding BDQ resistance. In a recent article in *ASM Case Reports,* Richard-Greenblatt et al. presented a case of treatment-emergent, cavitary, extensively drug-resistant (XDR) TB with a novel loss-of-function mutation in the *pepQ* gene conferring BDQ resistance (1). This commentary reviews BDQ-based regimen strengths while highlighting emerging resistance concerns illustrated by this case.

BDQ’s potency stems from inhibition of the proton pump c-subunit of mycobacterial ATP synthase, which affects both replicating and dormant bacilli (2). BDQ also degrades the transmembrane pH gradient, enhancing its bactericidal activity (3). *Mycobacterium tuberculosis* requires ATP synthase even under hypoxic, low-growth conditions, contributing to BDQ’s effect on nonreplicating persistent populations (2). BDQ’s long half-life (~5.5 months) and extensive tissue distribution maintain sterilizing concentrations in most compartments (4). In mice, BDQ-containing regimens showed superior sterilizing activity compared to standard therapy (5).

BDQ’s favorable antimicrobial and pharmacologic properties have fueled enthusiasm regarding its inclusion in all-oral, shorter-course DR-TB regimens. Over the last decade, numerous randomized controlled trials have demonstrated the efficacy and safety of all-oral, shorter-course BDQ-based anti-TB regimens (6–9). Based on these data, recent World Health Organization (WHO) and ATS/CDC/IDSA guidelines have endorsed the use of such regimens for DR-TB (10, 11).

**Fig 1:**
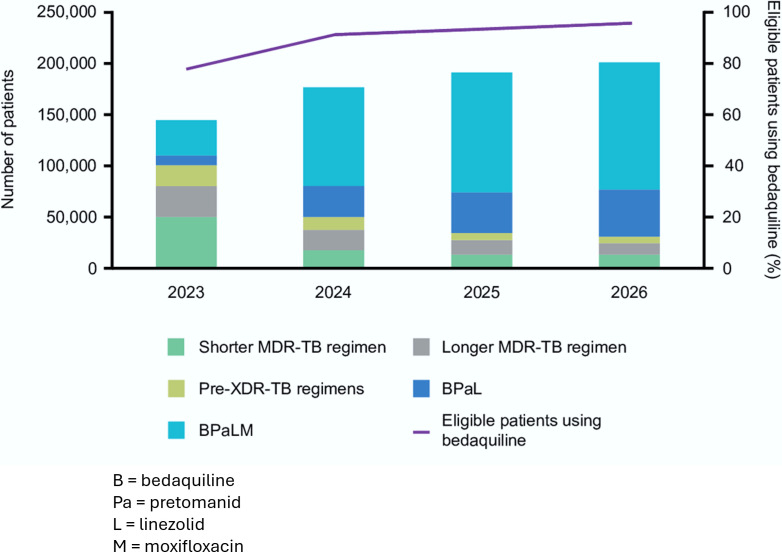
Projected use of bedaquiline, 2023–2026. B, bedaquiline; Pa, pretomanid; L, linezolid; M, moxifloxacin. Adapted from reference 14, previously published under a Creative Commons license.

Although the WHO reports a gradual decline in multidrug resistant (MDR) and rifampin-resistant (RR)-TB since 2015, an estimated 390,000 such cases occurred in 2024 (12). Treatment success rates are 71% vs 88% for MDR/RR-TB vs drug-sensitive TB, with 12% vs 3.5% mortality, respectively (12). For MDR-TB, treatment success rates and mortality rates have improved over time, largely due to the uptake of BDQ-based regimens (13). TB Alliance projections estimate that BDQ use in DR-TB regimens will increase from 145,000 to 203,000 patients annually from 2023 to 2026 (14). See Fig. 1.

Considering BDQ-based regimens’ vital importance in managing DR-TB, how has their uptake impacted the emergence of BDQ resistance? An early report from the French National TB program identified 4/209 (2%) MDR-TB isolates with increased minimum-inhibitory concentrations (MICs) to BDQ among patients treated between 2014 and 2015 (15). Mozambique reported 9% BDQ resistance in DR-TB isolates from 2015 to 2021, rising from 3% in 2016 to 14% in 2021 (16). A meta-analysis identified nearly 650 phenotypically BDQ-resistant isolates from 31 reports, with a pooled prevalence of 5.7%, with the highest prevalence reported for South Africa (17). South African reports show resistance rising from 3.8% (2015–2019) to 14% (2018–2022) (18, 19).

**Fig 2:**
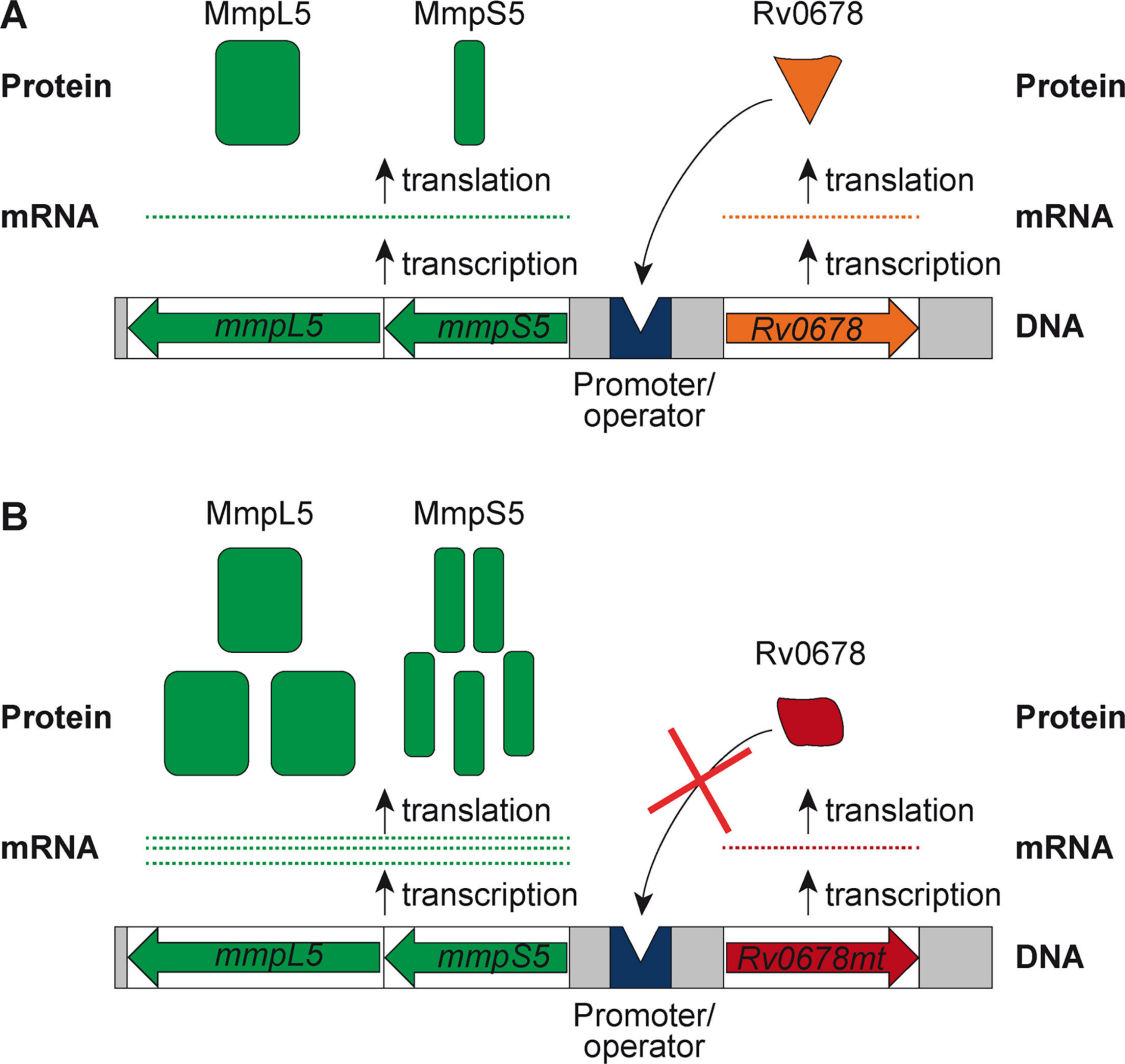
Mechanism of Rv0678 mutation mediated BDQ resistance. (**A**) Regulation of mmpS5 transcription by wild-type Rv0678 repressor. Rv0678 protein binds to the intergenic region location between Rv0678 and mmpS5, which contains the 10 consensus boxes of promoters for both Rv0678 and mmpS5. This prevents the RNA polymerase from starting transcription, resulting in the decrease in the expression of MmpS5, MmpL5, and Rv0678 proteins. In response to an unknown stimulus, the regulator detaches from DNA, and transcription can be resumed. (**B**) Lack of regulation in Rv0678 mutants. The strains carrying frameshift mutations in Rv0678 will not produce a functional repressor; thus, the transcription of mmpS5, mmpL5, and Rv0678 will be increased. If the mutation results in an amino acid polymorphism, the protein may still be functional, but with reduced DNA-binding ability depending on the location of the mutation. In either case, the final consequence will be an increase in the expression levels of the proteins MmpS5, MmpL5, and Rv0678. Adapted from reference 21, previously published under a Creative Commons license.

BDQ resistance arises via multiple genetic pathways. Early work from Swedish researchers identified 97 unique resistant isolates, with MICs ranging from 0.12 to 3.84 µg/mL, while wild-type isolates had a mean MIC of 0.03 µg/mL. Of the 53 resistant isolates sequenced, 15 harbored *atpE* mutations, the gene coding for the c-subunit of ATP synthase, the known BDQ binding site. These *atpE* mutations conferred substantially higher MICs than non-*atpE* mutations, with one, the A63P mutation, yielding an MIC >100 times that of wild-type isolates. Conversely, 38 isolates with elevated MICs had wild-type *atpE* and no identified mutations in other ATP synthase genes, suggesting that off-target mechanisms contribute to resistance (20). Janssen researchers later identified *Rv0678* mutations in non-atpE resistant strains, which upregulate the MmpS5-MmpL5 efflux pump and raise MICs; this effect could be partially reversed by efflux pump inhibitors verapamil and reserpine (21, 22). *Rv0678* mutations showed minimal fitness cost and can be selected *in vitro* by low-level BDQ or clofazimine (CFZ) exposure (21). See Fig. 2.

While prior reports have documented clinical, BDQ-resistant *M. tuberculosis* isolates harboring *pepQ* mutations, Richard-Greenblatt et al. identified a clinical isolate with a unique Glu 177 Stop mutation in *pepQ,* with associated elevated MICs to BDQ (MIC = 0.5 µg/mL [M. Richard-Greenblatt, personal communication]) (23). Earlier murine studies showed subtherapeutic BDQ/CFZ exposure selected *pepQ* mutants with four-fold MIC increases, independent of Rv0678 or *atpE* mutations. This gene encodes a proline-specific aminopeptidase. While co-incubation of isolates containing mutated *pepQ* along with efflux pump inhibitors attenuated MIC increases, no changes in mmpS5-mmpL5 transcription could be detected in such isolates. Given these observations, the precise mechanism linking *pepQ* loss-of-function to BDQ resistance remains unclear (24).

Work by South African investigators shed light on risk factors for both baseline and acquired BDQ resistance. They collected baseline (*N* = 2,023 specimens) and longitudinal specimens (*N* = 695 specimens) from clinical DR-TB cases between 2015 and 2019. Risk factors for baseline and acquired BDQ resistance included prior CFZ or BDQ exposure (odds ratio [OR] 7.1, 95% CI 2.3–21.9), fluoroquinolone resistance (OR 4.8, 95% CI 2.6–8.8), pre-extensive drug resistance (OR 4.2, 95% CI 1.7–10.5), and extensive drug resistance (OR 4.8, 95% CI 2.0–11.7) (18).

The Richard-Greenblatt et al. case report highlights the challenges of managing DR-TB. In this case, given limited alternative options, clinicians included CFZ in a holding regimen as they worked to obtain BDQ, and subtherapeutic CFZ levels may have contributed to the development of CFZ/BDQ resistance. The Richard-Greenblatt et al. case report also emphasizes the link between fluoroquinolone resistance and the emergence of BDQ resistance. The patient’s relapse strain tested fluoroquinolone-resistant, limiting the potency of the patient’s post-relapse regimen, and this may have facilitated the emergence of CFZ/BDQ resistance.

Even though BDQ has a large volume of distribution, its variable penetration into different compartments of pulmonary nodules/cavities may advance the development of resistance. Data have emerged indicating that granulomas’ caseous centers harbor high concentrations of nonreplicative persistent bacilli (25). Unfortunately, researchers have also demonstrated that BDQ concentrates in the cellular rim of TB-related granulomas but diffuses much more slowly into the necrotic caseum. This creates “two spatiotemporal windows” for subtherapeutic drug exposure—during the early phase of BDQ-based therapy as the drug diffuses into this compartment and after therapy ends—while the drug lingers due to its long half-life (26).

The limited availability of phenotypic drug susceptibility testing for BDQ complicates the interpretation of genetic susceptibility testing results. Modest MIC increases induced by *Rv0678* or *pepQ* mutations may have an uncertain clinical impact. Pooled BDQ, pretomanid, and linezolid regimen trial data showed that patients with baseline BDQ resistance had more frequent unfavorable microbiologic outcomes (3/12; 25%) vs those without resistance (6/185; 3.2%), but 9 of 12 with BDQ mutations still had favorable outcomes (27). Acquired resistance was rare, emphasizing the importance of potent multidrug regimens. Though more expansive whole-genome sequencing genotypic resistance testing approaches offer the benefit of identifying novel or unexpected resistance-associated variants beyond predefined targets, the use of large international databases such as the World Health Organization mutation catalog and the CRyPTIC Consortium serves to provide a critical framework for standardized interpretation of such emerging mutations (28).

The TB treatment community has responded to the threat of emerging BDQ resistance. The *in vitro* data that demonstrate efflux pump-mediated resistance may be attenuated by co-administration of efflux pump inhibitors have led to clinical trials to study this approach (29). In addition, several next-generation diarylquinoline anti-tuberculous agents are in development—with the goals of improving their early bactericidal activity, as well as their penetration into the caseous material (25, 30).

The Richard-Greenblatt et al. case illustrates several essential challenges related to the management of DR-TB. While BDQ-based regimens have allowed for improved outcomes and shorter courses of therapy, their wide uptake has been accompanied by the predictable emergence of resistance. The authors of this case report describe a novel *pepQ* mutation that confers BDQ resistance and, in doing so, they highlight several weaknesses of BDQ-based regimens: most notably how BDQ’s long half-life and variable caseum penetration may work in tandem to allow for resistance-inducing monotherapy in a compartment highly populated by dormant/persistent bacilli. The key vulnerability of BDQ/CFZ cross-resistance, made more pertinent in light of the typical delays in obtaining BDQ, also impacted the case report patient’s outcome, as did fluoroquinolone co-resistance. Despite the unfortunate outcome for the case report patient, the advance of knowledge from this case report and continuing work on new strategies and agents to treat DR-TB provide assurance that we fight on to eliminate the threat of DR-TB.
